# Do rotational shear-cushioning shoes influence horizontal ground reaction forces and perceived comfort during basketball cutting maneuvers?

**DOI:** 10.7717/peerj.4086

**Published:** 2017-11-23

**Authors:** Wing-Kai Lam, Yi Qu, Fan Yang, Roy T.H. Cheung

**Affiliations:** 1 Department of Kinesiology, Shenyang Sport University, Shenyang, China; 2 Li Ning Sports Sciences Research Center, Li Ning (China) Sports Goods Co., Ltd., Beijing, China; 3 Gait & Motion Analysis Laboratory, Department of Rehabilitation Sciences, The Hong Kong Polytechnic University, Hung Hom, Hong Kong

**Keywords:** Basketball shoe, Sidestep cutting, Lateral shuffling

## Abstract

**Background:**

Court shoe designs predominantly focus on reducing excessive vertical ground reaction force, but shear force cushioning has received little attention in the basketball population. We aimed to examine the effect of a novel shoe-cushioning design on both resultant horizontal ground reaction forces and comfort perception during two basketball-specific cutting movements.

**Methods:**

Fifteen university team basketball players performed lateral shuffling and 45-degree sidestep cutting at maximum effort in basketball shoes with and without the shear-cushioning system (SCS). Paired *t*-tests were used to examine the differences in kinetics and comfort perception between two shoes.

**Results:**

SCS shoe allowed for larger rotational material deformation compared with control shoes, but no significant shoe differences were found in braking phase kinetics during both cutting movements (*P* = 0.35). Interestingly, a greater horizontal propulsion impulse was found with the SCS during 45-degree cutting (*P* < 0.05), when compared with the control. In addition, players wearing SCS shoes perceived better forefoot comfort (*P* = 0.012). During lateral shuffling, there were no significant differences in horizontal GRF and comfort perception between shoe conditions (*P* > 0.05).

**Discussion:**

The application of a rotational shear-cushioning structure allowed for better forefoot comfort and enhanced propulsion performance in cutting, but did not influence the shear impact. Understanding horizontal ground reaction force information may be useful in designing footwear to prevent shear-related injuries in sport populations.

## Introduction

Cutting and shuffling movements accounted for more than 40% of the total typical movements in a basketball game (Brauner, Zwinzscher & Sterzing, 2012; Stacoff, Steger & Stussi, 1993). Among 11 typically tested basketball movements, cutting and shuffling movements were shown to present high horizontal ground reaction force (GRF) during the impact (McClay et al., 1994). Excessive horizontal GRF during the impact phase places large shear stress on the ligaments or other soft tissues of the lower limbs, and it is thought to be associated with non-contact anterior cruciate ligament injuries and ankle sprains (Cumps, Verhagen & Meeusen, 2007; McKay et al., 2001; Yu & Garrett, 2001). Furthermore, repetitive high shear forces may have caused discomfort and soft tissues injuries such as hyperkeratosis, calluses and blisters on the foot (De Luca, Adams & Yosipovitch, 2012; Spence & Shields, 1968; Sulzberger et al., 1966; Yavuz & Davis, 2010), which may negatively influence athletic performance. However, the design of the court shoe on shear force attenuation has received little attention among sport populations.

Court shoe designs have been shown to attenuate excessive impact force (Cavanagh, Williams & Clarke, 1981; Nigg, 2010), with its focus primarily on cushioning the vertical GRF (Nin, Lam & Kong, 2016; Zhang et al., 2005). Shear optimisation has not been widely explored in current basketball shoe design. Chan et al. (2013) invented a groove-type translational shear cushioning heel design to attenuate the horizontal GRF during braking phase of walking and running. However, such designs cannot be directly applied to basketball shoes due to two reasons. Firstly, cutting movements in basketball usually involve turning and thus both translational and rotational shear cushioning may need to be considered. Secondly, the shear cushioning device in the heel region may not compromise the horizontal force in the forefoot region during propulsion phase (Shimokochi et al., 2013). Sufficient shear/horizontal force is imperative for effective braking and propulsion to enhance performance (Lam et al., 2015; Shimokochi et al., 2013). Stefanyshyn et al. suggested that greater propulsion GRF was the key determinant in maximum sprinting, and vertical and long jumps (Stefanyshyn & Fusco, 2004; Stefanyshyn & Nigg, 1998). Thus, optimising horizontal GRF for sport orthotic footwear should consider both injury prevention (i.e., reduced shear force in braking phase) and sport performance enhancement.

A recently recognised Shear Cushioning System (SCS) concept (Shear Reduction, RedDot invention award 2017, Essen, Germany) was newly introduced to allow material deformation in rotational direction for shear cushioning, but scientific guidelines of the SCS footwear have yet been established. Additionally, a recent basketball study on plantar pressure (Lam, Ng & Kong, 2017) reported higher peak pressure and pressure time integral for medial and central forefoot compared with the heel region during sidestep cutting. Such findings suggested that the forefoot region might also be exposed to higher risks of injuries. Thus, the purpose of the present study was to examine the SCS effect on horizontal GRF and perceived comfort during the braking and propulsion phases of two typical cutting manoeuvres, i.e., 45-degree cutting and lateral shuffling. It is hypothesised that the SCS would reduce the horizontal shear during the braking phase, but increase shear during the propulsion phase in cutting movements. Better comfort perception was also expected from the basketball players with such a novel basketball shoe design.

## Materials and Methods

### Experimental shoe conditions

**Figure 1:**
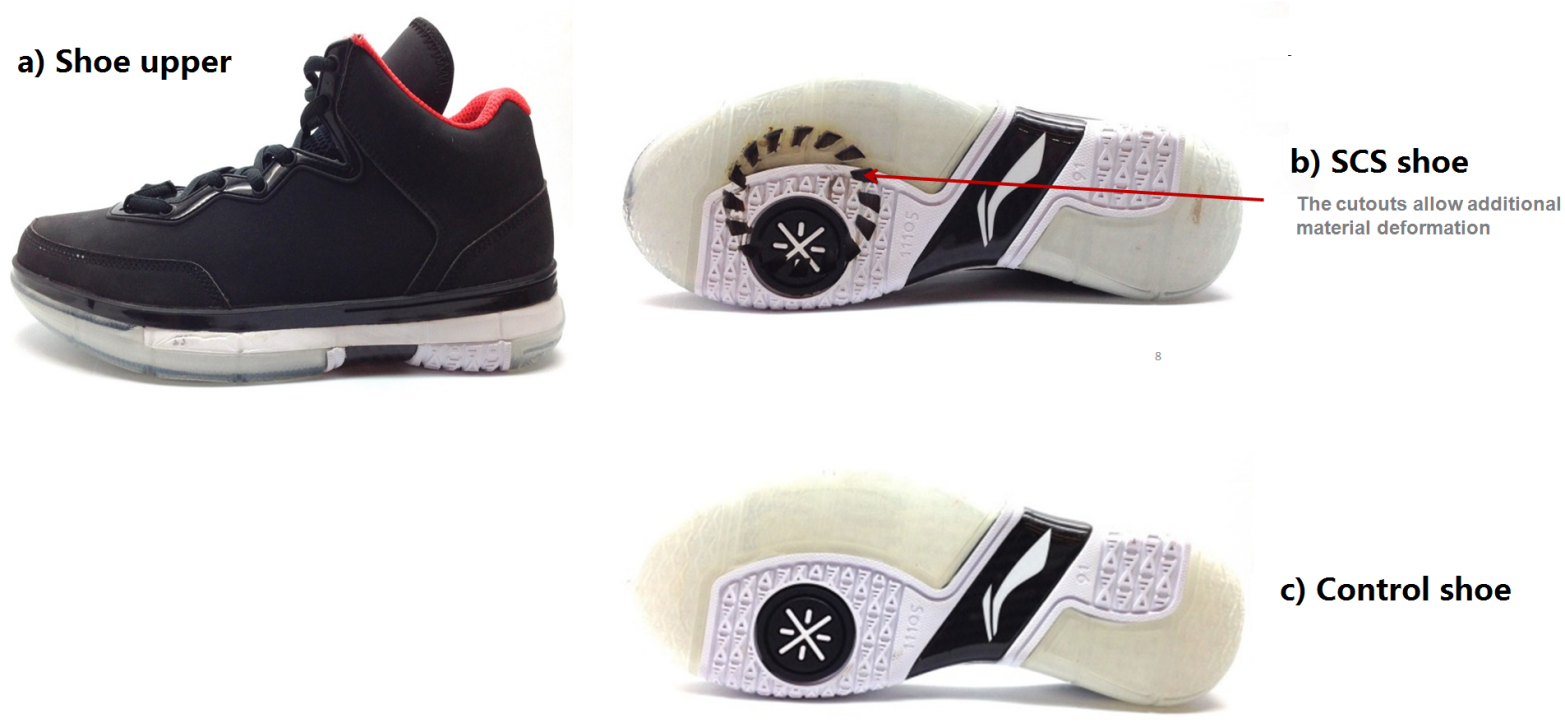
Basketball shoe conditions. (A) Shoe upper, (B) bottom view of SCS shoe and (C) bottom view of control shoe.

To examine this effect, two identical pairs of basketball shoes (Fig. 1, Li Ning Wade 1.0; Beijing, China) with and without SCS (SCS versus Control) were manufactured for this study. The control shoe was unmodified and retained its original specifications, identical to those available in the market. The SCS shoe was built with the identical cutout design at the forefoot region using the laser cutting machine (Fig. 1). The SCS was built at the medial forefoot region as this area was identified as the core supporting region of the foot during cutting movements based on the previous in-shoe shear force findings (Cong et al., 2014). This structural design was constructed to allow the material deformation in a rotational direction for shear cushioning, which was akin to the groove-type translational shear cushioning design and mechanism for a running shoe and walking insole (Chan et al., 2013; Lavery et al., 2005).

### Mechanical experiment

**Figure 2:**
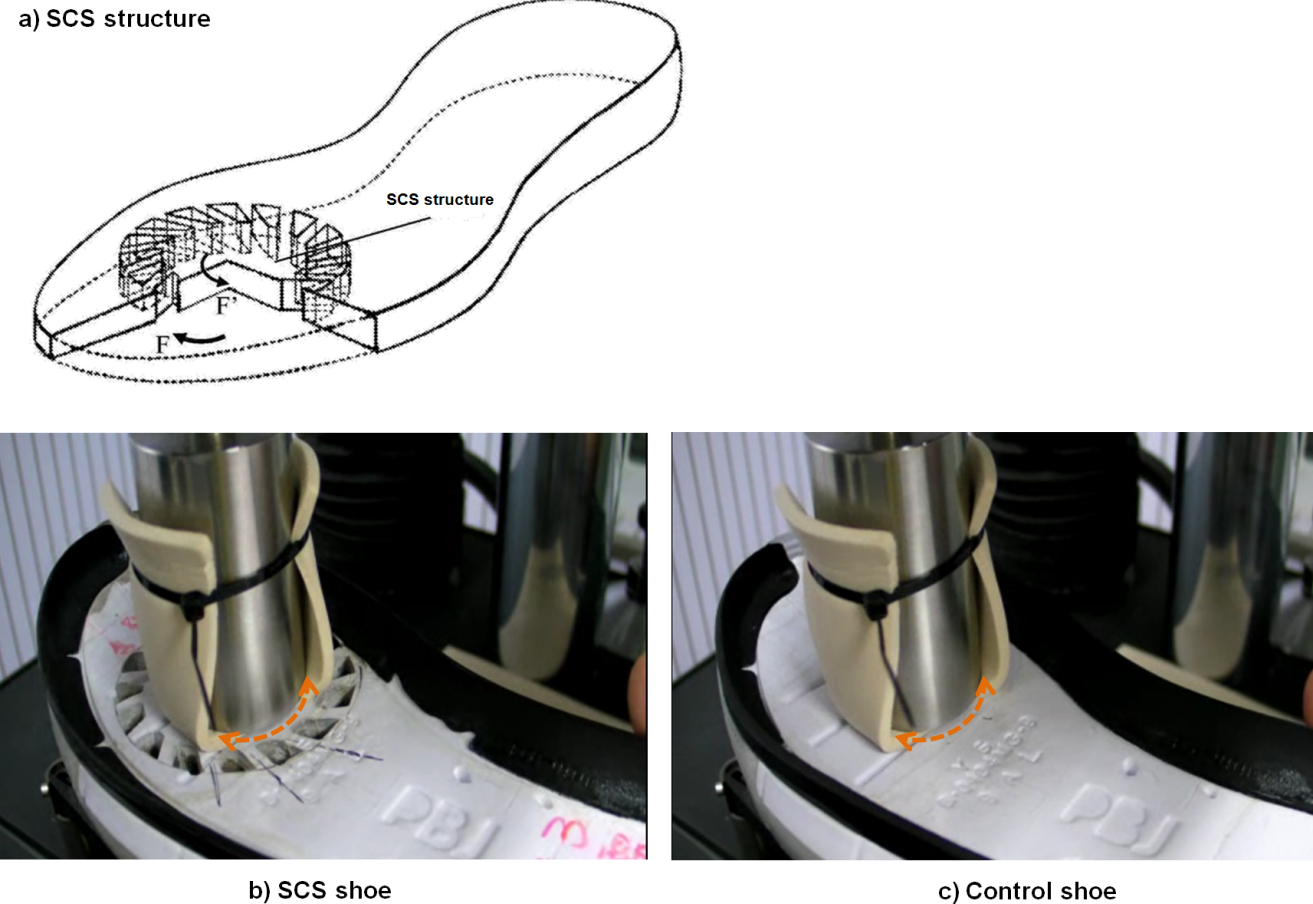
(A) The schematic shows a cross-section of the shear-cushioning system (SCS) with rotational force (F) and rotational reaction force (F’). (B) Mechanical rotation stiffness test on the shear-cushioning system (SCS) and (C) control shoes.

**Figure 3:**
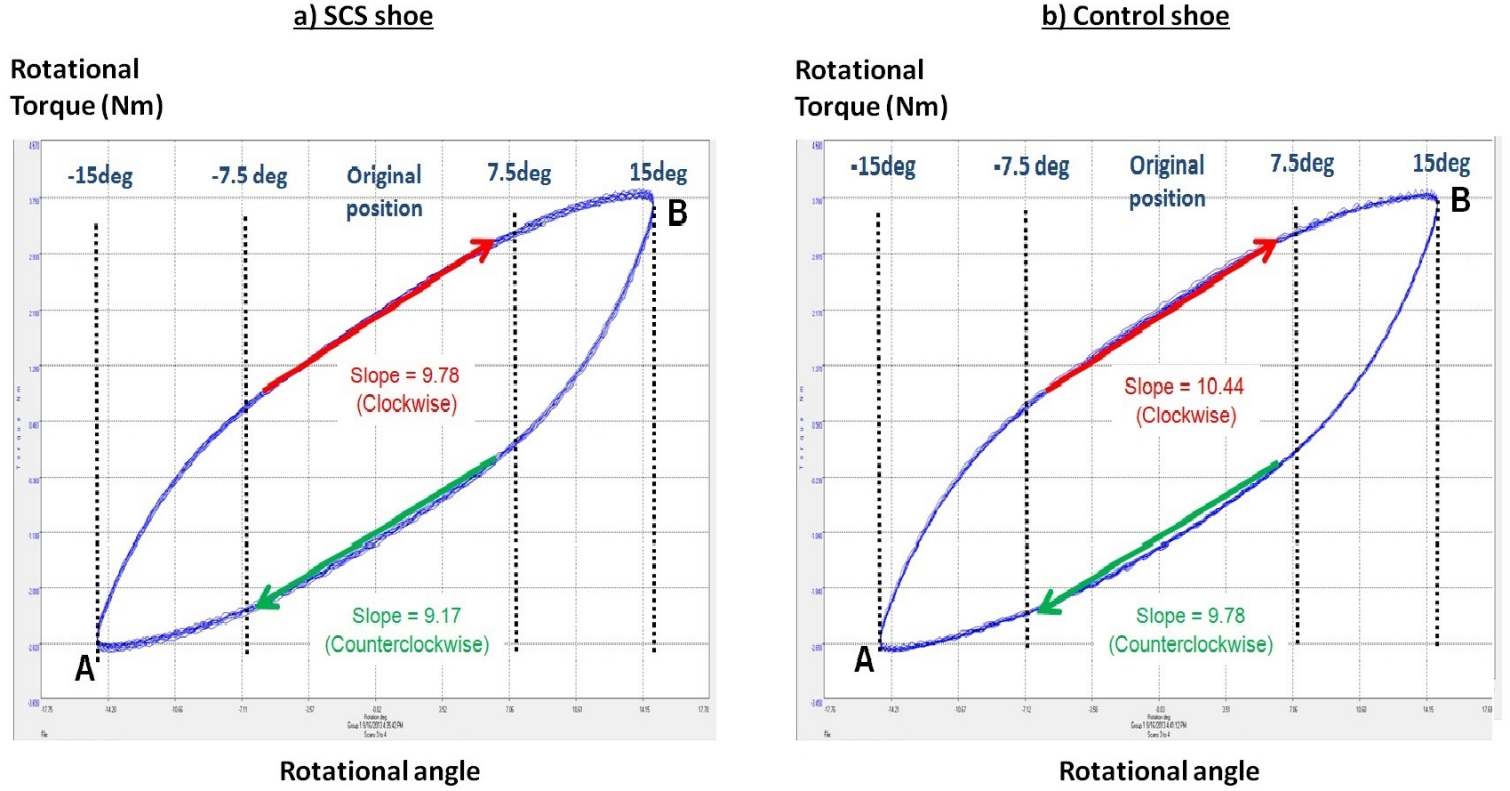
The mechanical loading cycles for (A) SCS and (B) control shoes.

The overall rotational stiffness of each test shoe was tested with a computer-aided dynamic material test machine (Bose ELF3550; Bose, Framingham, MA, USA), which allowed compression and rotation through controlled force or deformation. Firstly, the compression force of 250 N was applied at the corresponding SCS regions of both the SCS and control shoes (Fig. 2). Then, twenty consecutive rotation cycles from −15 to 15 degree/s were performed to obtain torque and angle data. This loading condition was specified from a previous study (Cong et al., 2014). Each test shoe was carefully aligned and secured with clamps throughout the test. The last five loading-unloading trials were averaged for the calculation of shoe rotation stiffness at a data sampling frequency of 1,000 Hz. The shoe rotational stiffness was defined as the linear region of the slope (−7.5 to 7.5 degrees) in the torque–angle plot (Fig. 3).

### Biomechanical experiment

#### Participants

Fifteen male university team basketball players (mean age = 21.7 ± 2.9 years; height = 1.78 ± 0.05 m; body mass = 70.6 ± 7.2 kg) were recruited. Average basketball experience and exposure were 6.4 ± 2.8 years and 11.9 ± 3.5 h per week, respectively. Sample size estimation was determined based on data reported by similar previous research (Lam et al., 2015) using G*Power software (http://www.gpower.hhu.de/en.html; Dusseldorf, Germany) with a beta of 0.8 and an alpha level of 0.05. All participants had normal foot arch and were free of any lower extremity injuries for at least six months prior to this study. Written consent was signed by participants prior to the start of the study. Ethical approval was granted by the Li Ning institutional review committee (IRB-2014BM015).

#### Testing procedures

**Figure 4:**
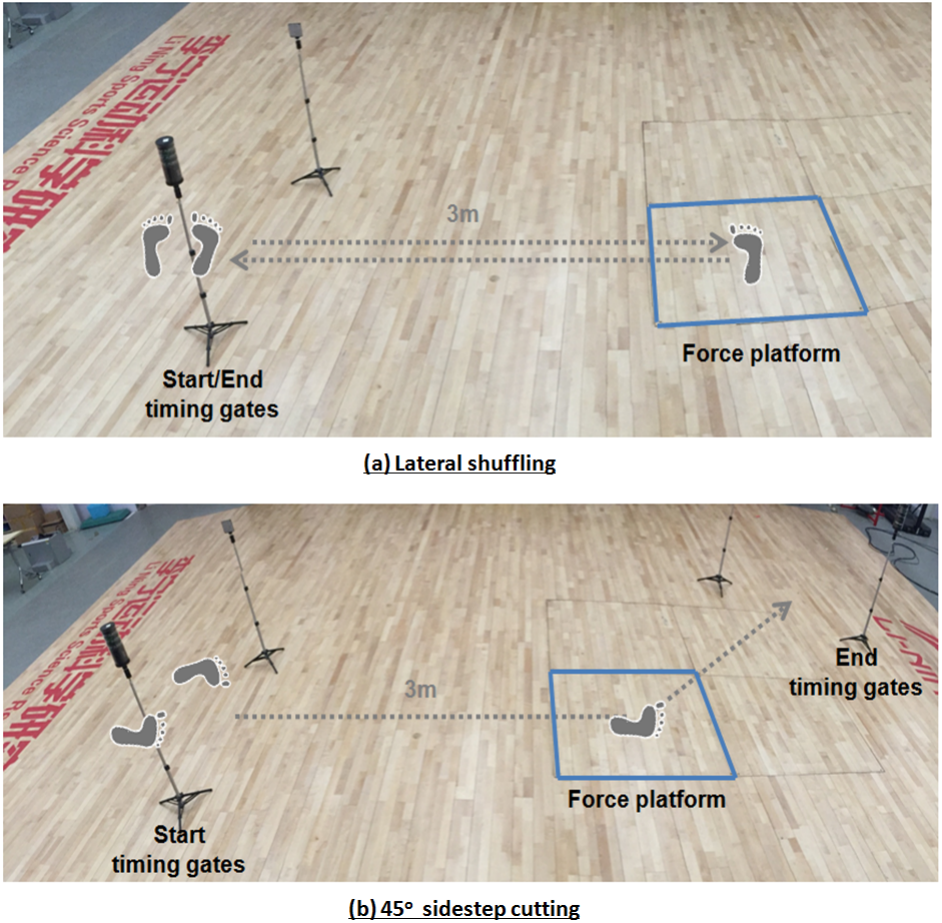
Movement tasks: (A) lateral shuffling and (B) 45-degree sidestep cutting. The footprints (grey color) indicate the foot placement on the force platform.

The lateral shuffling and 45-degree cutting protocols were referred to a previous study on basketball movements (Cong et al., 2014). In brief, the tests were administered on a standard basketball indoor court surface, which consists of an embedded 1.2 × 1.2 m wooden-top AMTI force platform (Advanced Mechanical Technology Inc, Watertown, MA, USA) at a sampling frequency of 1,000 Hz (Fig. 4). In order to detect the braking and propulsion phases, knee kinematics of the lower limb were analyzed. Reflective markers were firmly affixed at eight body landmarks: medial and lateral malleoli, medial and lateral epicondyles, the first, second, and fifth metatarsal heads, and the posterior midpoint of the heel counter (Cong et al., 2014). Two triad markers were attached at the shoe heel counter and tibia to record the trajectories of the shoe and shank segments, respectively. The marker trajectories were measured using an eight-camera motion analysis system (Vicon, Oxford Metrics, Oxford, UK) at 200 Hz. For lateral shuffling, the participants were instructed to perform lateral shuffling to the right with maximum effort. Each participant stepped onto the force platform with his right foot (the third step) and returned to the starting point as quickly as possible (Fig. 4A). For 45° sidestep cutting, each participant ran forward and stepped onto the force platform with his right foot from a starting point, 3m from the force platform. Thereafter, the participants performed maximal-effort cutting to the left at 45 degrees. They continued to run and passing through pylons positioned 1.5 m from the center of the force platform (Fig. 4B). A pair of photoelectric timing gates at heights of 1.1 m (Fusion Sport Smart Speed Timing Gates; Fusion Sport, Brisbane, Australia) was set-up at Start and End positions with a separated width of 3 m, as specified in the pilot study. The elapsed times between the trigger (Start) and exit (End) gates were measured as performance time. Prior to the actual data collection, participants familiarised themselves with the testing protocol including the placement of the right foot on the force platform in each of the test movements. A trial was only considered valid if the position of the right-leg cutting step was committed. Five successful trials were collected for each shoe and movement condition. Throughout the study, measures were taken to blind participants to the differences of the two shoes (e.g., only one pair of shoes was visible at any one time). The test sequence of shoe and movement conditions was randomised using an online program (http://www.random.org). Two-minute and five-minute resting periods were mandatory between trials and between shoe conditions, respectively.

Immediately after the test movements for each shoe condition, participants were asked to rate their forefoot cushioning perception of the shoe on a 150-mm Visual Analogue Scale (VAS) (not comfortable at all (0 mm) to most comfortable (150 mm)). The VAS is a common method to assess subjective perceptions of comfort in athletic shoes and allows for parametric data analysis (Lam, Sterzing & Cheung, 2011; O’Leary, Vorpahl & Heiderscheit, 2008).

#### Data analysis

**Figure 5:**
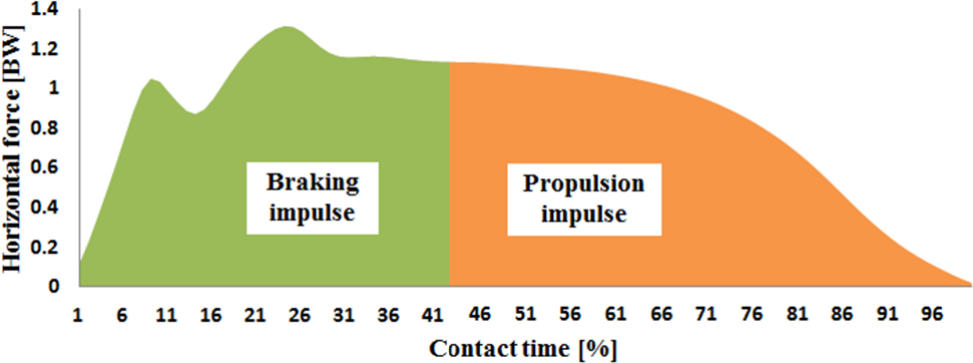
The definition of braking and propulsion impulses of horizontal force.

A spline interpolation was performed for minor missing marker trajectories using three frames of data before and after the missing data (Sigward & Powers, 2006). A fourth order Butterworth bidirectional filter with a cut-off frequency of 12 Hz was used to smooth the kinematic data (Nigg et al., 2009). The kinetic data was filtered using a fourth order Butterworth filter at 100 Hz and normalised to body mass (Nigg et al., 2009). The marker trajectories were identified manually using Vicon Clinical Manager Software (Oxford Metrics Ltd, Oxford, UK) and then transferred into Visual3D software (C-Motion Inc, Ontario, Canada) to define the peak knee flexion angle for the determination of braking and propulsion phases (Lam et al., 2015). The instance of foot contact and take-off were defined as when the vertical GRF first exceeded 10 N (foot contact) and below 10 N (take-off). Horizontal impulses were calculated as the time integrals (i.e., area for respective braking and propulsion periods) under the ground reaction force curves (Fig. 5). Peak resultant horizontal GRF forces and impulses in both braking and propulsion phases were calculated for this study.

Descriptive mechanical rotational stiffness was reported for the tested shoes. All statistical analyses were performed using SPSS 21.0 (IBM Corp., Armonk, NY, USA). Paired *t*-tests were used to examine the difference in the horizontal GRFs, impulses and subjective comfort between shoe conditions. Global alpha was set at 0.05. In order not to over-rely on the statistical tests, the effect size (Cohen’s d) was calculated using PASS (version 13; NCSS Statistical Software, Kaysville, UT, USA).

## Results

### Shoe mechanical characteristics

The mechanical evaluation showed that rotational stiffness of SCS shoes (clockwise direction: 9.78 ± 0.14 Nm/rad and counterclockwise direction: 9.17 ± 0.16 Nm/rad) were about 6% smaller than the control shoes (clockwise direction: 10.44 ± 0.06 Nm/rad and counterclockwise direction: 9.78 ± 0.11 Nm/rad), indicating that SCS shoe would allow for larger rotational material deformation.

### Biomechanical and comfort variables

For lateral shuffling, there was no significant difference in total foot contact time (Cohen’s d = 0.24; *P* = 0.35), horizontal GRFs and impulses (Cohen’s d = 0.12 to 0.23; *P* > 0.34), and forefoot perception (Cohen’s d = 0.23; *P* = 0.31, Table 1) between test shoes.

**Table 1:** Foot contact time, kinetics and comfort level (mean and standard deviation) during lateral shuffling in shoes with SCS and control.

	SCS	Control	Cohen’s d	*P* value
Total foot contact time (s)	0.53 (0.09)	0.51 (0.08)	0.24	0.35
Peak braking horizontal force (BW)	1.31 (0.24)	1.27 (0.23)	0.17	0.54
Braking horizontal impulse (BWs)	0.37 (0.04)	0.36 (0.04)	0.23	0.35
Peak propulsion horizontal force (BW)	0.93 (0.09)	0.94 (0.08)	0.12	0.63
Propulsion horizontal impulse (BWs)	0.17 (0.02)	0.17 (0.02)	0.20	0.34
Forefoot comfort perception	9.70 (2.32)	9.18 (2.28)	0.23	0.31

For 45° cutting, a greater propulsion horizontal impulse was found in players in shoes with SCS than the control shoes (Cohen’s d = 0.30; *P* = 0.05, Table 2). It may explain better perceived comfort of forefoot when participants wore shoes with SCS than the control shoes (Cohen’s d = 0.65; *P* = 0.012, Table 2). On the other hand, there was no significant difference in completion time and total foot contact time (Cohen’s d = 0.14, 0.33; *P* > 0.35), nor braking and propulsion GRFs and braking impulse (Cohen’s d = 0.05, 0.30; *P* > 0.53) between the tested shoes (Table 2).

**Table 2:** Foot contact time, completion time, kinetics and comfort level (mean and standard deviation) during 45-degree cutting in shoes with SCS and control. Significant *p*-values (*P* < .05) are shown in bold.

	SCS	Control	Cohen’s d	*P* value
Total foot contact time (s)	0.24 (0.03)	0.23 (0.03)	0.33	0.15
Completion time (s)	1.05 (0.07)	1.04 (0.07)	0.14	0.72
Peak braking horizontal force (BW)	1.61 (0.27)	1.58 (0.26)	0.11	0.53
Braking horizontal impulse (BWs)	0.22 (0.02)	0.21 (0.02)	0.05	0.85
Peak propulsion horizontal force (BW)	1.23 (0.21)	1.24 (0.21)	0.05	0.69
Propulsion horizontal impulse (BWs)	0.07 (0.02)	0.06 (0.02)	0.30	**0.05**
Forefoot comfort perception	9.90 (1.97)	8.39 (2.71)	0.65	**0.01**

## Discussion

Sport footwear designers should consider shoe cushioning, comfort and performance, which are the most important footwear features for basketball players (Brauner, Zwinzscher & Sterzing, 2012; Losito, 2008). Changing the structural design might alter the material deformation properties (i.e., loading response between ground and shoe/foot complex) during impact activities and thereby influence the footwear comfort perception (Nin, Lam & Kong, 2016), cushioning (Chan et al., 2013; Zhang et al., 2005), and performance (Stefanyshyn & Nigg, 1998). Horizontal GRF may be another risk factor of cutting related injuries but receive limited attention in basketball; the SCS footwear was designed with a shear cushion interface to allow larger rotational deformation and thereby avoid internal shear forces on the foot. The present study sought to examine the effects of the novel basketball shoe design on the horizontal GRF and perceived comfort during basketball-specific cutting manoeuvres. The results indicated that even though SCS shoes would allow for larger rotational material deformation (∼6%) compared with control shoes, the SCS prototypes did not alter the GRF and impulses during the braking phase in any of the test movements, which did not support our original prediction. Perhaps a larger size of the cutouts, a different shape of cutouts and/or softer midsole material may allow a larger degree of material deformation so as to examine if shear cushioning could be plausible in basketball.

Instead, the results suggest that the SCS prototypes can allow for better forefoot comfort perception (+18%) and higher horizontal impulse (+17%) in 45-degree cutting movement (*P* < 0.05). A plausible mechanism for higher impulse is that the structural design (see Fig. 2A) might allow larger rotational deformation for accumulating/storing potential energy during braking (F denoted in Fig. 2A), and release the energy during propulsion (F’ denoted in Fig. 2A). Another possible mechanism is that the asymmetric cutouts might allow stronger muscular forces to be generated for push-off. From a minimum energy dissipation concept (Stefanyshyn & Fusco, 2004), the shoe structure (such as midsole hardness or inserted plate) might minimise energy loss at the metatarsophalangeal joint during push-off or take-off action. For future studies, the structural deformation should be quantified from the bottom view using a grass-cover (transparent) force platform, and electromyography should be applied to understand the underlying muscle activation strategy or onset pattern (Landry et al., 2009) before a viable conclusion can be made.

The cushioning properties of a shoe have been suggested to be associated with subjective comfort (Nin, Lam & Kong, 2016). Based on the perceptual ratings, participants are expected to modify their movement patterns to avoid high impacts in different activities (Nigg, 2001). Habitually rearfoot strike runners would change their strikes to a midfoot/forefoot striking pattern when running in barefoot conditions (Lieberman et al., 2010). Basketball players would change their knee joint kinematics when landing from a higher landing height (Zhang et al., 2005). The optimal pathway hypothesis (Nigg, 2001) might explain a better forefoot comfort with greater shear impulse in SCS shoes, as the SCS might have provided better comfort and allowed the execution of the optimal pathway of individuals. The present results indicated that, compared to the control shoes, players wearing shoes with SCS displayed better forefoot comfort perception in 45-degree cutting but not in the lateral shuffling movement. It was possible that the 45-degree cutting a incorporated more turning components, making it easier for players to assess and give the comfort rating more effectively. To confirm this speculation, future studies should examine the relationship between the thresholds of GRF and the perception rating in high-demand tasks such as cutting and shuffling.

One limitation would be that we did not examine the effects across participants with foot types (Chuckpaiwong et al., 2008) or anthropometry (Nin, Lam & Kong, 2016), and these factors may have influenced the GRF measurements. Furthermore, individual differences in movement strategy and physical condition could lead to non-significant findings for some kinetic data. According to a maximum dynamic hypothesis (Markovic & Jaric, 2007), the muscular system of the lower limbs is designed to optimise dynamic output when loaded with the individual anthropometry characteristics. In the future, more investigation should be carried out on players across different foot types, anthropometry and training levels before a valid recommendation can be made. Although the exact mechanism of rotational shear-reduction structure has yet to be explored from the present findings, it could be of interest to further optimise the footwear structure design that has reduced excessive horizontal stress in braking phase without negatively affecting athletic performance in propulsion phase.

## Conclusion

Although the rotational shear-reducing shoe structure did not show direct benefit to attenuate horizontal force loadings in braking phase of cutting movements, the players wearing the shoes with this structure displayed better forefoot comfort perception and greater propulsion impulses in 45- degree cutting with maximum-effort. This implies the possibilities for a footwear rotational shear cushioning/comfort structure without compromising cutting performance. Understanding horizontal GRF data may be useful in designing orthotic footwear for sport populations.

##  Supplemental Information

10.7717/peerj.4086/supp-1 Data S1 All raw data

## Additional Information and Declarations

### Data Availability

The following information was supplied regarding data availability: The raw data has been provided as Data S1.
